# A New Technique for Percutaneous Nephrolithotomy Using Retrograde Ureteroscopy and Laser Fiber to Achieve Percutaneous Nephrostomy Access: The Initial Case Report

**DOI:** 10.1089/cren.2018.0079

**Published:** 2019-08-30

**Authors:** Carlos A. Uribe, Hugo Osorio, Johana Benavides, Carlos H. Martinez, Zachary A. Valley, Kamaljot S. Kaler

**Affiliations:** ^1^ Division of Urology, Hospital Pablo Tobón Uribe, Medellín, Colombia.; ^2^ Department of Urology, Clínica CES, Medellín, Colombia.; ^3^ Department of Urology, CES University, Medellín, Colombia.; ^4^ Division of Urology, Pablo Tobón Uribe, Medellín, Colombia.; ^5^ Department of Urology, University of California, Irvine, California.; ^6^ Endourology and Robotic Surgery, Department of Surgery, Southern Alberta Institute of Urology, University of Calgary, Alberta, Canada.

**Keywords:** percutaneous nephrolithotomy, ureteroscopy, laser, nephrostomy access

## Abstract

***Background:*** Percutaneous nephrolithotomy (PCNL) serves as the gold standard minimally invasive procedure to remove large renal stones. The puncture is made from the skin to the chosen calix under fluoroscopic guidance, although this remains a challenging technique. We describe the initial case of retrograde holmium laser acquired nephrostomy access.

***Case Presentation:*** In this study, we present the case of a 48-year-old woman with right renal colic with imaging revealing a 2.6 cm staghorn stone. With institutional approval, we performed a new technique utilizing retrograde access with a flexible ureteroscope and a holmium laser fiber to achieve nephrostomy access for PCNL in the prone position. With the ureteroscope confirmed in the desired calix, the ureteroscope and laser fiber were aimed and fired toward the flank and thus creating a subcostal nephrostomy tract. PCNL was then carried out per standard of care lithotripsy techniques utilizing the holmium laser.

***Conclusion:*** In this initial case, percutaneous retrograde laser access allowed for desired caliceal nephrostomy access under direct vision.

## Introduction and Background

Percutaneous nephrolithotomy (PCNL) has been accepted as the gold standard minimally invasive procedure to remove large renal stones. Typically, the needle puncture for nephrostomy access is made in an antegrade manner from the skin into to the targeted calix under fluoroscopic vision. In 1983, Hunter and colleagues presented their experience of a retrograde nephrostomy technique performed in 30 patients.^1^ The technique featured a puncture wire that was placed within a retrograde catheter and steered into the desired calix where it then punctured through the renal parenchyma, muscle, fascia, and skin. The catheter was then passed over it and the newly created tract would be able to be dilated for PCNL. In the author's initial experience, the procedures were attempted in 83% of patients. The aforementioned access device would later be commercialized as the Lawson retrograde nephrostomy wire puncture set (Cook Medical, Bloomington, IN); however, despite being readily available, the technique had not become widely accepted.

At the present time, percutaneous nephrostomy access is still typically achieved in an antegrade manner with ultrasonic and/or fluoroscopic guidance. Despite being the standard of care at most centers, this technique is often associated with a steep learning curve and most urologists are unable to do the procedure without the assistance of an interventional radiologist. With the widespread use of the flexible ureteroscope and recent advances in technology allowing for better vision and more precise movements, ureteroscopy-assisted retrograde nephrostomy access (UARN) has been described.

In this study, we present our initial experience using this novel holmium laser UARN modality for PCNL in a patient suffering from a staghorn calculus.

## Presentation of Case

**FIG. 1.**
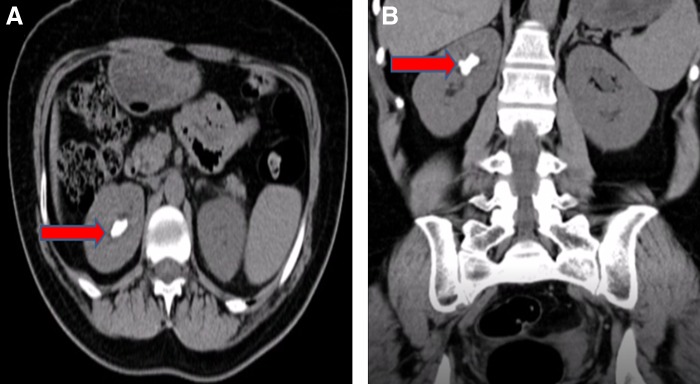
CT scan showing a 2.6 cm staghorn stone with 826 HU density in the right kidney (*red arrow*). **(A)** Axial view. **(B)** Coronal view.

The following case describes a 48-year-old woman who initially presented with right renal colic and was otherwise healthy. A CT scan showed a 2.6 cm staghorn stone with a density of 826 HU in the upper pole of right kidney (Fig. 1). Her serum creatinine level was 0.7 mg/dL and her urinary tract infection was diagnosed and treated before the PCNL took place, with preoperative sterile urine culture for confirmation.

### Technique

**FIG. 2.**
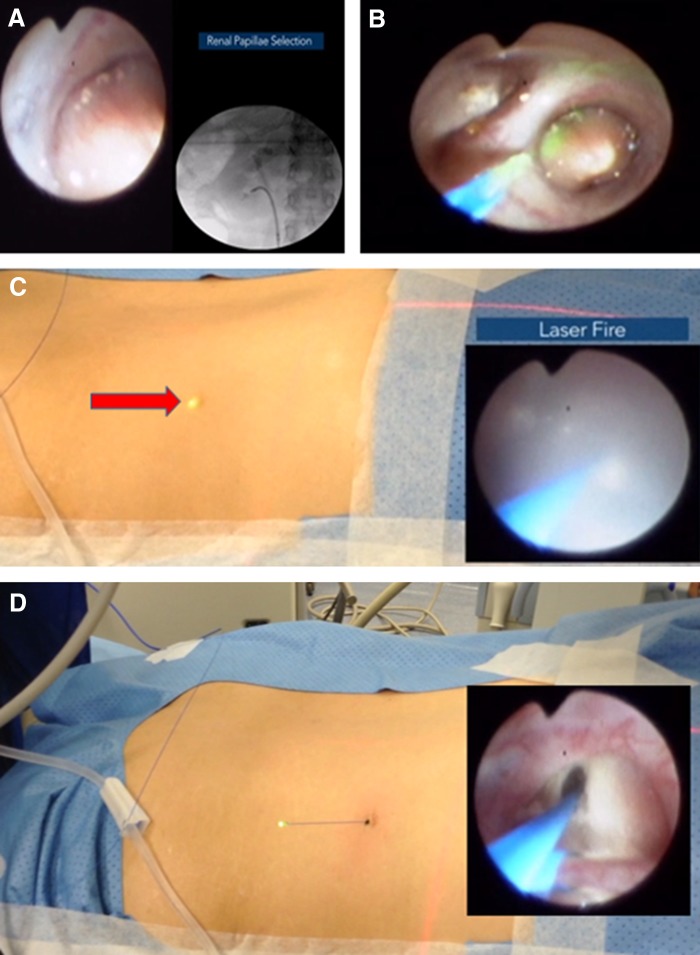
Percutaneous renal access achieved through passage of a 270 μm laser fiber. **(A)** The most posterior calix is identified using pyelography and retrograde air is injected into the renal space to act as fluoroscopic contrast. **(B)** The 270 μm retrograde holmium laser fiber is passed into the selected calix and the laser is fired continuously through the papilla. **(C)** The glow of the laser can be observed on the patient's exterior through the skin (*red arrow*) as the laser advances through the flank. **(D)** The laser fiber is observed while emerging from the skin without any bleeding.

The patient was administered general anesthesia and oriented in the prone split leg position. First, a cystoscope was introduced to identify the right ureteral orifice. Then, a sensor guidewire was passed under fluoroscopy guidance until it was confirmed in the ureteropelvic region. A flexible ureteroscope was then inserted into the kidney over the wire followed by pyelography and retrograde air injection to select the most posterior calix for nephrostomy access. A 270 μm holmium:YAG laser wire (Olympus, Hamburg, Germany) with 1 J and 10 Hz was used to puncture the papillae in the inferior calix to create a subcostal tract passing through the renal parenchyma, renal capsule, flank muscles, fascia, and finally the skin where the laser wire was observed and exteriorized (Fig. 2).

**FIG. 3.**
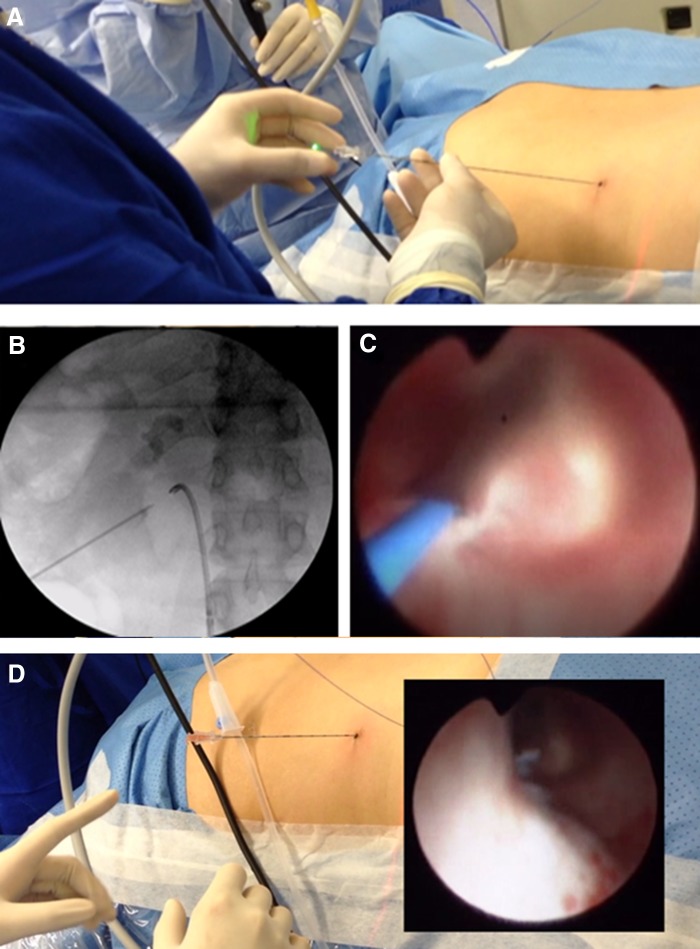
Insertion of the nephrostomy needle through the previously created nephrostomy tract. **(A)** The laser fiber was used as a guide to pass the needle over it. **(B)** Fluoroscopic image showing the nephrostomy puncture tract. **(C)** Direct endoscopic view of nephrostomy needle insertion, which was guided by the laser fiber (*blue wire*). **(D)** The laser fiber is removed and clear urine is observed to confirm access into the calix.

The complete puncture procedure was done under direct endoscopic vision and care was taken at all times to avoid bleeding. Using the externalized laser fiber as a guide, an 18-gauge needle was passed into the desired calix. With the needle in place, the laser fiber was removed, and clear urine was observed flowing from the needle, which confirmed proper access in the selected calix (Fig. 3).

**FIG. 4.**
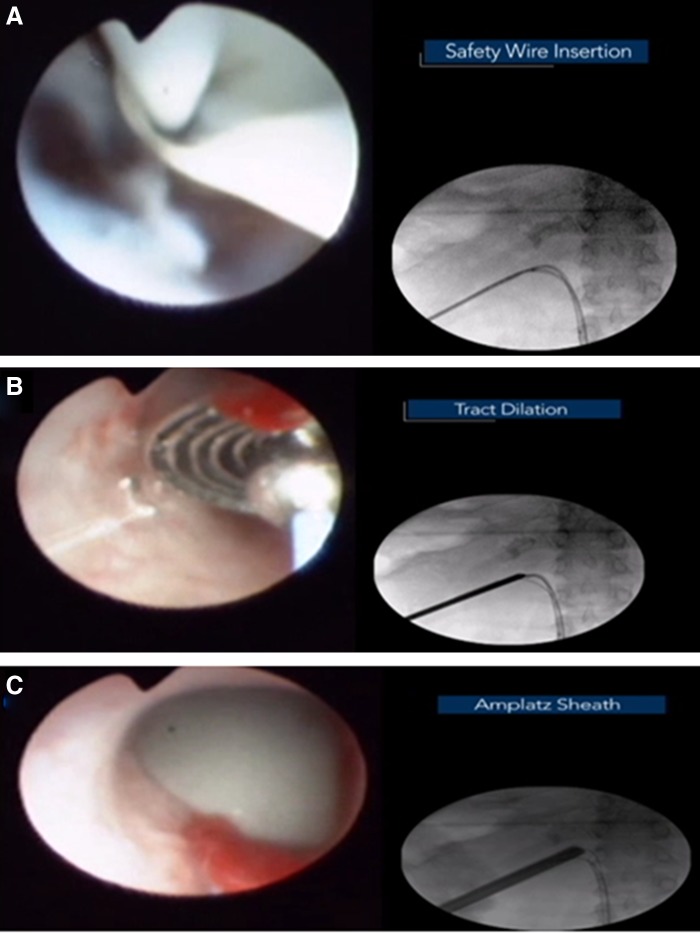
Nephrostomy access dilation. **(A)** Insertion of 10F double-lumen catheter to pass a secondary safety wire. **(B)** Dilatation of the tract was performed with sequential metal dilators. **(C)** Amplatz sheath insertion.

A guidewire was then inserted into the renal pelvis and the needle was removed. The guidewire was redirected in an antegrade manner into the bladder by means of the ureteroscopic NCircle^®^ tipless nitinol basket (Cook Medical). After this, a second guidewire was similarly inserted into the bladder in an antegrade manner. With the wires in place, the nephrostomy tract was dilated to 30F using Alken metallic sequential dilators before inserting a final Amplatz sheath to conclude percutaneous access (Fig. 4).

With the use of a 26F rigid nephroscope (Karl Storz, Tuttlingen, Germany), the staghorn calculus was observed and further inspection of the renal cavities was performed. A flexible nephroscope (Olympus) was introduced into the posterior calix for lithotripsy using the 270 μm holmium:YAG laser. The residual fragments of the calculus were extracted with the 2.2F NCircle basket. Finally, a 6F Polaris™ Double-J stent (Boston Scientific, Natick, MA) was inserted in an antegrade manner and an 18F Foley catheter was inserted through the tract to serve as a nephrostomy tube.

### Operative results

The total operative time was 180 minutes with no intra- or postoperative complications. The length of stay was 2 days. The patient's pain was treated with paracetamol and opioids. On the first postoperative day, the nephrostomy tube was plugged with no additional imaging. On the second postoperative day, the nephrostomy tube was removed without evidence of bleeding or pain and the incision was sutured. Two hours after the nephrostomy tube was removed, the Foley catheter was removed and the patient was discharged from the hospital.

## Discussion and Literature Review

The variable level of success during a PCNL relies heavily on proper nephrostomy placement within the kidney. For a complicated procedure that deals with large burden, such as a staghorn calculus, optimal nephrostomy placement is critical for complete and efficient ablation of the stone without the need for additional accesses. Although this aspect of the procedure is important for improved surgical outcomes, the standard antegrade nephrostomy access is difficult for the practicing urologist and often requires the expertise of an experienced interventional radiologist. Given that the use of radiologists to establish percutaneous access during PCNL is extremely common in modern urologic practice, it has been shown that urologist-gained access is more likely to result in improved stone-free status.^2^ This finding should encourage urologists to create their own nephrostomy access, but many are unable to do this because of the difficulty and steep learning curve of traditional antegrade nephrostomy access.

In an attempt to reconciliate the issue of access difficulty, urologists created the technique of retrograde access for PCNL in 1983, but because of technological limitations of the time, the procedure never gained popularity. Recently, drastic improvements to ureteroscopic technology has allowed for this idea of retrograde access. With the ability to guide the access needle under direct vision in a retrograde manner, the learning curve has been shown by Yin and colleagues to be exceedingly shallow while simultaneously allowing for lower fluoroscopy times during the PCNL procedure.^3^ We further modified this with the use of a laser fiber.

The potential benefits of using a holmium laser for access is the laser's characteristics of coagulation, cutting, efficacy, and widespread use. The laser itself has thermal properties that would assist in reducing bleeding without unnecessary peripheral tissue damage because of its short range. The laser itself is widely used in contemporary urologic practices and can be the primary mode of lithotripsy during PCNL. Thus, using the laser for the additive purpose of nephrostomy access would reduce extraneous costs by reducing the number of instruments needed, while also increasing efficiency because of reduced instrument exchange.

To increase the accuracy of our tract creation, the most posterior calix under direct vision was chosen with simultaneous retrograde contrast and air bubble introduction into the collecting system as a guide. The preoperative noncontrast abdominal CT showing the kidney, bowel, and other organs' locations were additionally studied to avoid complications (i.e., retrorenal organs). Although our initial experience with the 270 μm laser fiber was satisfactory for the puncture, the possibility of fiber bending must be considered. However, we believe that the “cutting” properties of the laser reduce the required force needed to advance the wire through the flank since the laser pulse carves the access path, unlike the Lawson wire that relies entirely on force. Nonetheless, it might be reasonable to use a 360 μm laser fiber in the future cases to prevent an errant path during the creation of the tract. We have found that the thinness of the laser fiber results in the possibility of fiber breakage. However, this can be resolved intraoperatively by removing the laser fiber at the skin and retracting the internal aspect. In addition, future utilization of a radiopaque laser fiber would serve as a benefit by having simultaneous fluoroscopic guidance during the puncture, and potentially reduce the risk of complications.

A potential drawback of this modality occurs if the ideal upper pole calix is a stone-bearing calix that cannot be accessed retrogradely by the ureteroscope. In this case, the stone of interest would be fragmented until the papilla is exposed for proper retrograde laser access. We believe that this technique would not result in a significant difference in access for a typical noncomplete staghorn stone, as the laser fiber would already be on standby and inserted in the ureteroscope. Future studies regarding this situation are warranted, as this initial case was carefully selected to avoid this problem. In addition, we believe that striking an intercostal vessel during intercostal access with this technique, in theory, may have a slightly greater risk than the standard antegrade nephrostomy technique, as standard nephrostomy allows for improved external puncture placement. However, one could also injure those vessels during placement of nephrostomy sheath with a rotating action. In fact, the coagulative effects of the laser may reduce the amount of bleeding when compared with striking an intercostal vessel with a Lawson wire or standard nephrostomy needle. Nevertheless, further studies regarding intercostal vessel puncture and bleeding severity are required, as this initial case was carried out with a subcostal access tract without any significant peripheral damage.

Despite our very limited initial experience, we believe the ease of this novel technique may serve as an alternative to antegrade access and may result in more urologists achieving their own accesses during PCNL in the future with broader studies.

## Conclusion

Percutaneous retrograde laser puncture allows for the accurate localization of the ideal nephrostomy calix under direct vision, reduced bleeding risk, and decreased radiation for the patient and the surgeon. In addition, using the laser fiber as needle makes the puncture much easier for the urologist, which allows for less reliance of the radiologist to achieve access.

## Abbreviations Used

CT: computed tomography
HU: Hounsfield units
PCNL: percutaneous nephrolithotomy
UARN: ureteroscopy-assisted retrograde nephrostomy access
